# Induced Pluripotent Stem Cell Therapies for Cervical Spinal Cord Injury

**DOI:** 10.3390/ijms17040530

**Published:** 2016-04-09

**Authors:** Vanessa M. Doulames, Giles W. Plant

**Affiliations:** Stanford Partnership for Spinal Cord Injury and Repair, Department of Neurosurgery, Stanford University School of Medicine, 265 Campus Drive Stanford, California, CA 94305, USA; vanessad@stanford.edu

**Keywords:** spinal cord injury, cervical, iPSC, induced pluripotent stem cell, embryonic stem cell, intraspinal transplantation

## Abstract

Cervical-level injuries account for the majority of presented spinal cord injuries (SCIs) to date. Despite the increase in survival rates due to emergency medicine improvements, overall quality of life remains poor, with patients facing variable deficits in respiratory and motor function. Therapies aiming to ameliorate symptoms and restore function, even partially, are urgently needed. Current therapeutic avenues in SCI seek to increase regenerative capacities through trophic and immunomodulatory factors, provide scaffolding to bridge the lesion site and promote regeneration of native axons, and to replace SCI-lost neurons and glia via intraspinal transplantation. Induced pluripotent stem cells (iPSCs) are a clinically viable means to accomplish this; they have no major ethical barriers, sources can be patient-matched and collected using non-invasive methods. In addition, the patient’s own cells can be used to establish a starter population capable of producing multiple cell types. To date, there is only a limited pool of research examining iPSC-derived transplants in SCI—even less research that is specific to cervical injury. The purpose of the review herein is to explore both preclinical and clinical recent advances in iPSC therapies with a detailed focus on cervical spinal cord injury.

## 1. Introduction

Spinal Cord Injury (SCI) places a formidable emotional, physical, and financial burden on the United States. Annually, the incidence of survived SCI is estimated at 12,500 cases leading to an estimated total of 276,000 with over half occurring at the cervical level. The causes of general SCI tend to be accident or violence related, however, the weight-bearing and flexible nature of vertebrae at the cervical level make it particularly susceptible to injury [1,2]. Some cervical-specific causes of injury include direct and indirect military-based injuries (via combat or through weaknesses in tactical armor design) [3,4,5,6,7] and lifestyle choices (such as sedentary lifestyles) leading to structural degradation of the cervical spine [8,9]. Ironically, advancements in modern healthcare have also been influential in the increasing incidence of survived cervical SCI; improvements in emergency medicine have led to better survival rates immediately following injury [10] while improvements in preventative care have led to the steady increase of an aging population and therefore age-associated injuries, degeneration, and weaknesses of the cervical spine [11,12,13].

Less than 1% of patients with SCI leave the hospital with a full neurological recovery; lengthy bouts of hospitalization, outpatient medical care (such as rehabilitation), and the need for full or part-time caretakers are often required. Coupled with injury-related motor deficits, they are often unable to participate in the workforce. In fact, by 10 years post-injury, only around 28% of patients are employed. Medical attention and injury-related lifestyle changes are financially overwhelming; over the course of a lifetime, they can accrue up to $4.5 million in costs directly associated with SCI. Overall, SCI costs the nation over $20 billion in direct and indirect injury-related expenses [1,2].

**Figure 1:**
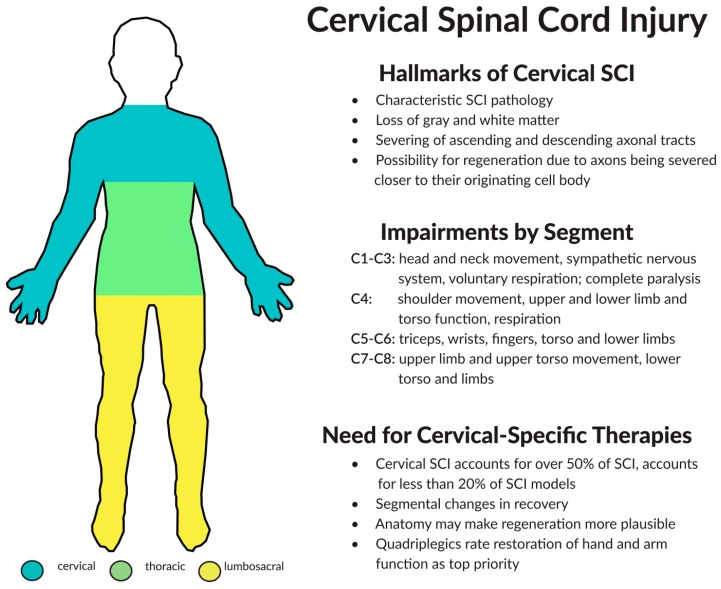
Clinical deficits, segmental differences, and the need for cervical-specific therapies for Spinal Cord Injury (SCI) within the human.

Despite survival rates increasing, quality of life still remains drastically poor with cervical SCI patients encountering a gradation of respiratory and upper motor limb dysfunction, full or partial paralysis, neuropathic pain, extensive financial obligations, lack of personal independence, and resulting significant lifestyle adjustments. Therapies to improve quality of life and restore function, even partially, would make a huge impact and help ease the physical, financial, and emotional burden placed on cervical SCI patients and their caretakers (Figure 1).

## 2. Components of Cervical Spinal Cord Injury (SCI)

### 2.1. Pathophysiology

**Figure 2:**
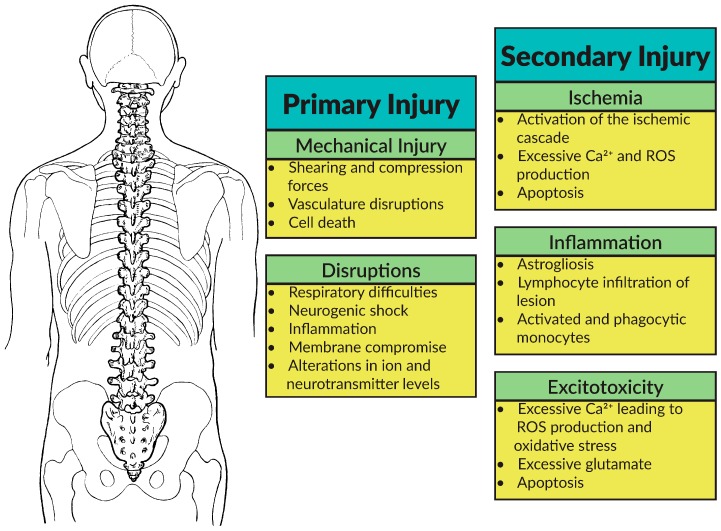
The Spinal Cord Injury (SCI) Cascade is comprised of both a primary and secondary component that ultimately results in ischemia, inflammation, and reactive oxygen species (ROS)-based excitotoxicity.

SCI is characteristically comprised of two phases that, while distinct, still maintain some level of overlap—primary and secondary injury (Figure 2). The primary phase occurs immediately at the time of injury and is directly caused by gross physical trauma to the spinal cord. There are four major categories of primary injury: impact plus persistent compression, impact alone with transient compression, distraction, and laceration or transection. During primary injury, the delicate spinal cord tissue is mechanically compromised due to shearing and compression forces either by direct contact or inadvertently through manipulation of the vertebrae. This initial trauma leads to mechanical injury, disruptions in vasculature, respiratory difficulties, neurogenic shock, inflammation, membrane compromise, alterations in ion and neurotransmitter levels, and ultimately sets the stage for the secondary phase of injury [14,15,16].

While primary phase-associated damage leads to an immediate and often serious impairment of neurological function, secondary phase typically dictates the full magnitude of injury. There are approximately 25 well established mechanisms that constitute the secondary phase of injury, yet much remains to be elucidated as to how these pathways converge and play upon each other to determine the full manifestation of injury [17,18]. This biochemical cascade activates the ischemic pathway, which leads to neurotransmitter imbalances that underlie excitotoxicity. Other consequences such as inflammation and immune responses, swelling, and neuronal apoptosis also occur [19,20,21,22,23,24,25].

Mammalian SCI triggers large zones of necrosis at the site of injury, leading to cystic cavitation, creating gaps in the circuitry, and preventing communication with rostral centers along the central nervous system (CNS) into the brain. Axons within the spinal cord fail to regenerate after injury and retract towards the soma, with the majority stopping close to the injury lesion border. Overall, this results in a change to normal motor, sensory, and autonomic function depending on injury location. In humans, high cervical injuries are the most severe and often result in full paralysis with respiration, speaking, and bowel function also affected. Lower cervical injuries lead to partial paralysis, and gradations in respiratory, bowel, and arm and hand function. Thoracic injuries are dependent on level with injury to higher levels primarily affecting the full trunk and legs and lower levels affecting the bowels and legs. Injuries at the lumbosacral level lead to impairments in voluntary bowel control and partially affect function in the hips and legs, but patients are often able to retain limited or full walking ability.

### 2.2. Regeneration and Plasticity

Prior studies have suggested that the adult mammalian CNS does not regenerate, predominantly due to the inability of neurons to regenerate axons through the inhibitory milieu of the glial scar and injured spinal cord lesion [26]. Despite this, some degree of functional recovery is often seen, likely due to reorganization of spared circuitry and heavily dependent on the axonal sprouting of both spared lesioned and intact fibers [26,27,28]. Experimental evidence has shown that axonal regeneration and functional recovery can be influenced and promoted via the usage of synergistic therapies such as the addition of neurotrophic growth factors [29,30,31,32], the deletion of inhibitory factors typically associated with the lesion [33,34,35], and rehabilitation regimens and physical activity [36,37,38]. Despite this, the innate regenerative capabilities of the CNS are often overwhelmed by the extent of injury and functional behavior is drastically affected.

**Figure 3:**
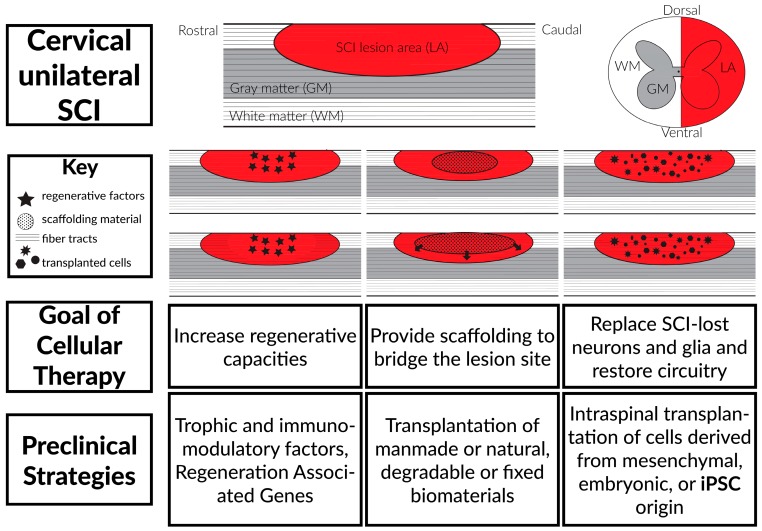
Current SCI therapeutics are calibrated to increase the regenerative capacities of the lesion site, provide a bridge through the lesion site to promote reconnection of rostral and caudal central nervous system (CNS), and to replace SCI-lost neurons and glia via transplantation of mesenchymal, embryonic, and induced pluripotent stem cells (iPSCs).

The immense damage following SCI results in pathophysiological heterogeneity and therefore requires complex therapeutic interventions that are engineered to address the individual components of the problem. Preclinical and clinical cell transplantation therapies may provide a solution to this by seeking to ameliorate existing damage and prevent further exacerbation by increasing innate regenerative capacities, providing scaffolding to bridge the lesion site and by replacing SCI-lost neurons and glia via intraspinal transplantation (Figure 3) [39,40,41,42,43,44,45].

The goal of this latter approach is to repair connectivity along the CNS by overcoming glial scar formation, thus serving as a cellular relay system or via promoting neurite regeneration [46]. In recent decades, the therapeutic promise of replacing neurons and glia by intraspinal transplantation has gained significant interest and some have eventuated to clinical trials. Numerous pre-clinical experiments have been developed including peripheral nerve bridges, Schwann cells, olfactory glia, mesenchymal stem cells and neural stem cells [47,48,49,50,51,52,53,54]. Initially, embryonic neural tissue was grafted into SCI lesion sites leading to some restoration of anatomy and function. The heterogeneity and source of the implanted tissue made direct “bench to bedside” translatability of this approach difficult and unlikely to gain required FDA approval as a clinical treatment [55]. Embryonic neural progenitor cells provide an alternative means to addressing cell loss and support following SCI, yet also add an additional ethical barrier that may endanger its applicability as a viable clinical treatment [56,57]. Therapies derived from human induced pluripotent stem cells (iPSCs) are in their infancy, but overcome many of the barriers of alternative existing cellular approaches. They provide the significant additional benefit of patient-matching, therefore surmounting the need for immunosuppression between host and donor cells. The review herein explores both preclinical and clinical recent advances in iPSC therapies targeting cervical SCI.

### 2.3. Considerations for Treating Cervical SCI

Cervical level injuries account for the majority of SCI, however, over 80% of studies have used various mid-thoracic injury models [58]. Although gross similarities in pathophysiology exist between the levels, there are key distinctions that underlie the need for cervical-specific models and therapeutics.

Anatomically, injuries at the cervical level are characterized by severing of the ascending and descending axonal tracts of the white matter and substantial cell loss within the gray matter. At the thoracic level, injuries tend to involve white-matter damage. This translates to distinct segmental differences in motor function at the cervical level that simply do not apply to thoracic injuries. Clinically, restoring function in a mid to lower cervical injury in even a singular segment would translate to greater patient independence. One study reported that restoration of function at the 5th and 6th cervical (C5/C6) level would allow a patient to independently type, eat, drink, wash, shave, dress their upper body, have more control over their wheelchair, and be able to transfer themselves from a chair to a bed or a car [59]. Restoration of a singular segment at the thoracic level would not translate to the same magnitude of functional recovery.

Furthermore, axonal tracts descending from rostral centers within the CNS are damaged closer to their originating soma in cervical *versus* thoracic SCI. There is substantial evidence that long descending axons rarely regenerate in injuries at the mid-thoracic level or lower but can at the cervical level [60,61,62]. Interestingly in mammalian quadruped models of SCI, animals that receive thoracic injuries are often able to regain some level (if not all) of locomotion, presumably due to the presence of a central pattern generator in the lumbar segments and the restructuring of propriospinal circuitry [63,64]. Supporting this was a key study in which decerebrate cats received a full spinal transection in the lower thoracic region and were still able to perform basic walking motions when electrophysiologically stimulated, thus suggesting that the supraspinal tracts originating in the motor cortex may not even be imperative to basic function [65,66,67]. In contrast, in rat models of cervical SCI, unilateral hemisection injury in the lower cervical levels leads to the irreversible loss of fine motor control of the forepaws and substantial motor deficits in the biceps and triceps brachii muscles [68,69,70,71]. Moreover, during reach and grab behavioral assessments, the recruitment pattern for proximal and distal pairs of antagonist muscles showed highly disorganized activation patterns [72].

Survivors of cervical SCI are faced with quadriplegia and all the sensorimotor deficits that accompany it. In a survey distributed to the SCI community and composed of 681 responses, the top priority of quadriplegics was restoration of hand and arm function—even above locomotion [73]. Restoration of function at a singular cervical segment could mean the difference between independence and full-time caretakers. Based on anatomical and functional differences between spinal levels, therapies that target regeneration of the descending tracts at the cervical level may be worth pursuing, further indicating that thoracic SCI models are not always fully translatable towards cervical SCI.

## 3. Stem Cell Transplantation Therapies

### 3.1. Background

Stem cells are naturally occurring, undifferentiated cells that have the unique ability to both divide to produce more stem cells for self-renewal, and, differentiate into specific cell lineages (potency) under particular physiological conditions. Stem cells act as a repair and turnover system in both the developing embryo and adult, with the additional role of differentiating into all germ lines for organ formation within the embryo. Whereas self-renewal is essentially the same *in vivo* for cells of embryonic or adult somatic origin, potency is variable. Embryonic stem cells (ESCs) are harvested from the inner cell mass of blastocysts within four to five days post fertilization whereas adult stem cells (also termed mesenchymal stem cells; MSCs) are predominantly harvested from the bone marrow, adipose tissue, and occasionally the umbilical cord tissue and blood, molars, and several other locations. ESCs from the blastocyst are pluripotent—capable of differentiating into all three germ lines whereas MSCs are multipotent and are limited to lineages of the mesodermal layer. The ability to harvest and culture naturally-occuring stem cells and the subsequent ability to differentiate them towards specific phenotypes has instigated a surge in advancements in developmental biology, disease pathogenesis, and regenerative medicine.

It is beyond the scope of this review to detail all the *in vitro* and *in vivo* capabilities and progress using both ESCs and MSCs as this has already been accomplished by several elegant reviews [74,75,76,77,78,79,80,81,82,83,84,85]. The following sections briefly overview preclinical and clinical uses of stem cells in cervical SCI.

### 3.2. Mesenchymal Stem Cells (MSCs)

MSCs are commonly classified and identified by their ability to adhere to plastic, their expression of CD73, CD90, and CD105, the lack of expression of CD14/CD11b, CD79, CD19, CD34, CD45, and HLA-DR surface markers, and their multipotent ability to differentiate into mesodermal lineages *in vitro* [85,86,87,88,89,90]. The distribution of MSCs in a variety of adult somatic sources, their ability to respond to cues produced by tissue damage based on their association with the vasculature, the potential for autologous transplants, their trophic and immunomodulatory secretion capabilities, their ease and rapidity in harvesting and expansion, and minimal risk of tumorigenicity have made them potential candidates for stem cell transplantation following SCI [91,92,93,94,95,96,97,98,99,100,101,102,103,104]. Furthermore, MSCs transplantation has been tested in clinical trials looking at neurological, cardiovascular, and immunological disease and has been deemed safe [105]. MSCs are multipotent, indicating their restriction towards mesodermal lineages. The ability to differentiate beyond this capacity towards neuronal and glial lineages is a hotly debated topic, in part due their weak expression of neuronal marker NeuN (neuronal specific nuclear marker) and neurotrophic/neuroprotective properties [106,107,108,109]. Despite this, several groups have demonstrated that MSCs are incapable of a “true” neuronal fate [75,89,108].

MSCs are most commonly derived from the bone marrow of the iliac crest and, as such, represent the bulk of studies examining MSCs transplantation following cervical SCI. Human umbilical cord blood also provides a rich source of adult stem cells and has therefore generated much interest in stroke, traumatic brain injury, and SCI. Generally, MSCs transplantation following SCI leads to amelioration of inflammation, apoptosis, and glial scarring in conjunction with increased axonal regrowth, angiogenesis, and tissue sparing in both cervical [110,111,112,113,114,115,116,117] and thoracic models [106,118,119,120,121,122,123,124,125,126,127,128,129,130,131,132]. In clinical trials of thoracic SCI, autologous bone marrow transplants delivered either intravenously or intra-arterial in patients were found to be safe. Although motor and sensory function did improve, it is inconclusive whether therapeutic benefit was due to the transplants [118]. In one clinical case study utilizing MSCs derived from umbilical cord blood demonstrated improved sensory perception and movement in the patient’s hips and slight regeneration in and caudal to the lesion within 41 days transplantation following SCI [133].

### 3.3. Embryonic Stem Cells (ESCs)

ESCs are harvested from the blastocyst and half the capacities for both self-renewal and pluripotency. In addition, the characterization of 59 lines of demonstrated similar expression patterns of SSEA3, SSEA4, TRA-1-60, TRA-1-81, GCTM2, GCT343, CD9, Thy1 (also known as CD90), tissue-nonspecific alkaline phosphatase and class 1 HLA, and developmental genes *Nanog*, *Oct4*, *TDGF1*, *DNMT3B*, *GABRB3* and *GDF3* that maintain the potency state and help reduce unwanted differentiation [134]. In the field of SCI, current research studies using ESCs have been promising, in part due to the versatility in potency and decades of cell culture experience.

Embryonic neural tissue grafts became a popular endeavor during the 1970s and 1980s and transplantation within animal models showed positive outcomes in axonal projections between graft and host tissue, the secretion of glial proliferation-inhibiting factors, and demonstrated integration of host astrocytes into the donor graft. Despite this, the tissue did not survive or integrate well in large lesion areas (such as with a complete transection) or if the age of the donor graft was inappropriate, and required a rich vascular surface [135,136]. The culturing of ESCs began in the early 1980s using murine sources demonstrated *in vitro* survivability without the support of fibroblast feeder layers but required leukemia inhibitory factor (LIF) [137,138]. Overall this led to earlier xeno-free expansion and characterization than human sources, which originated in the late 1990s and were found to rely on fibroblast growth factor (FGF) to retain their pluripotency in culture [139,140,141].

The pluripotent capacities of ESCs also make them an extremely versatile and attractive option in studying neurodegenerative pathophysiology following injury or illness. Unlike MSCs that are limited to cell fates within a mesodermal lineage, ESCs can be driven towards ectodermal lineages, or more specifically, neural subtypes. *In vivo* transplantation of undifferentiated ESCs often leads to teratoma formation, so there has been a strong push to develop high-caliber differentiation protocols to mitigate the risk of tumor formation and create specialized cell populations that enhance the therapeutic potential within the heterogeneous niche of the spinal cord lesion [142]. ESCs driven towards and neural lineage and grafted provide an innovative approach in solving the loss of connectivity following SCI. Neural stem and progenitor cells have been found to overcome the inhibitory milieu of an SCI lesion site by promoting axon growth across the injury, remyelinating host axons, and facilitating synaptogenesis between host and donor; their use in cellular transplantation strategies therefore have significant potential to improve measurable functionality in SCI models [143,144,145]. A substantial portion of ESCs differentiation protocols supports the selection of astrocyte and oligodendrocyte populations over neurons in part due to glial proliferation, and has been found to assist in improving myelination, weight support, and gait in a model of thoracic SCI [58,146,147,148,149,150,151,152,153,154,155,156,157]. Other directed differentiation protocols have led to the derivation of neural progenitor cells and additional neuronal phenotypes such as spinal motor neurons, cholinergic, serotonergic, dopaminergic, noradrenergic, medium spiny striatal neuronal, and deep cortical pyramidal [156,157,158,159,160,161,162,163,164,165,166,167] for use in thoracic SCI models as well as other neurodegeneration-based pathologies.

In models of cervical SCI, the transplantation of whole fetal spinal cords or fetal neural progenitor cells taken from brain and spinal tissue has been associated with supraspinal growth, axonal projection and growth, differentiation into all three neuronal lineages, long distance cell migration, and improvement in skilled forelimb function [168,169,170,171,172]. Since 2013, human fetal-derived tissue and stem cell transplantation for cervical and thoracic SCI has eventuated into several clinical trials in which safety and efficacy are being assessed, based on promising results seen in thoracic SCI and amyotrophic lateral sclerosis models [173,174,175,176,177].

In contrast to transplantation of derivations of fetal tissue, several groups examined the transplantation of ESCs cultured towards different lineages within cervical SCI. Sharp and colleagues (2010) transplanted human ESC-derived oligodendrocyte progenitor cells seven days following a severe midline contusion in a rat model of cervical SCI [151]. Transplantation led to a decrease in lesion size, white and gray matter sparing, preservation of host motor neuron pools, minimal migration of transplanted cells, differential changes in spinal cord gene expression of neurons, growth factors, apoptosis, and inflammation, and increased forelimb function. Sun and colleagues (2013) also utilized human ESC-derived oligodendrocyte progenitor cells four months post cervical irradiation injury in a rat model and witnessed markedly less demyelination and improved forelimb locomotion. Furthermore, transplanted cells were shown to differentiate into mature oligodendrocyte phenotypes that expressed myelin basic protein [178].

In a change of direction, Rossi and colleagues (2010) drove human ESCs towards a progenitor motor neuron phenotype that *in vitro* were shown to be Olig 1/2^+^, Tuj1^+^, and Hb9^+^ , had functional glutamate receptors, and could innervate human and rodent muscle [179]. When transplanted following a cervical contusion in a rodent model of SCI, the cells displayed variation in differentiation pathways and phenotypes dependent on location, reduced lesion size, increased survival and growth of host neurons, and correlated with improved performance in motor tasks.

### 3.4. Potential Drawbacks of Adult and Embryonic Stem Cell Therapies

Current literature clearly demonstrates that stem cell transplantation therapies have shown promise in treating SCI, however, there are certain potential drawbacks that need to be considered and addressed when assessing this line of therapeutics for clinical purposes.

MSCs can easily be harvested from abundant adult somatic sources, are quick to culture *in vitro*, and are genomically stable. They have distinctive immunomodulatory and growth factor secretion capabilities, and clinically allow for the use of autografts thus circumventing the need for immunosuppression. Despite this their multipotency limits their ability to replace SCI-lost cells. They are less plastic than ESCs and there is evidence that MSCs do not persist and integrate following transplantation; overall, functional recovery post treatment remains limited to modest improvements [180,181,182,183,184,185,186].

ESCs are pluripotent, thus allowing for the manipulation of specific neuronal lineages and phenotypes following SCI. They rapidly proliferate in culture and have demonstrated significant functional recovery and amelioration of SCI pathology following transplantation. However, there are some concerns that potentially limit their use in clinical applications. ESCs are harvested from the blastocyst or from fetal-derived tissue, and thus are subject to ethical constraints and limited availability [187]. Their pluripotent capacity also introduces the risk of tumor formation following transplantation of undifferentiated cell populations, although this can be curbed by the use of more maturated cell types [188,189,190,191,192,193]. In culture, prolonged passage also leads to karyotypic abnormalities and genetic amplification that can contribute to oncogenesis [194,195,196]. Additionally, ESC allograft transplantation results in the need for immunosuppression, which can further confound the complicated and integral role the innate immune response plays following SCI.

Transplanting stem cells and their derivatives to treat SCI is a logical approach as they are capable of replacing cell phenotypes lost during injury, can induce powerful regenerative and immunological changes within the host tissue, and can potentially bridge the inhibitory environment of the lesion so that circuitry can hopefully be restored. The ideal cell type would be able to combine the advantages of both MSCs and ESCs while bypassing their individual pitfalls.

## 4. Induced Pluripotent Stem Cell-Derived Therapies

### 4.1. Induced Pluripotent Stem Cells

Induced pluripotent stem cells (iPSCs) offer a promising alternative to the potential drawbacks of embryonic and adult somatic stem cells. Whereas MSCs are limited in their potency, and ESCs are harvested via the manipulation of a pre-implantation embryo, iPSCs are a type of pluripotent stem cell that can be created from adult somatic cells by harvesting these cells and “reprogramming” them towards a stem cell state via the transduction of pluripotency genes.

The discovery that mature cells could be reprogrammed to pluripotency was initially pioneered by the Yamanaka laboratory in 2006. Therein it was demonstrated that iPSCs could be induced from mouse fibroblasts (and later using adult human fibroblasts) via retroviral delivery of Oct3/4, Sox2, c-Myc, and Klf4 without the need for Nanog [197,198,199,200]. Since then, iPSCs technology has progressed and multiple processes can be used to induce pluripotency in somatic cells. Viral transduction is easy to use, reproducible, yields iPSCs efficiently, and is controlled. However there is an increased risk of insertional mutagenesis and transgene reactivation, incomplete slicing, and clone-to-clone variation [201,202,203]. Reprogramming factors can also be fused to cell-penetrating peptides or introduced through plasmids, which requires no genomic modification but is also a very slow and inefficient process [204]. Finally, iPSCs can be induced via mRNA introduction. It is a highly efficient (and faster) method, requires no genomic modification, and is safe due to the transient nature of mRNA; however, repeated transfections are typically required [205,206].

Initial work suggested that iPSCs and ESCs were transcriptionally different possibly due to differential promoter binding by the reprogramming factors, variances in genetic background, or studying small set numbers [207,208,209,210]. There is a large body of research though that concludes that ESCs and iPSCs are molecularly and functionally equivalent after accounting for differences in genetic background in that they share the same morphology, gene markers and expression, mitochondrial properties, and tumorigenicity [209,210,211,212,213,214,215,216,217].

The ability to create pluripotent stem cells from adult somatic cells circumvents some of the difficulties of using ESCs and MSCs–ethical barriers are minimized, cells can be driven towards any lineage, and, in the case of potential transplants, cells can be harvested directly from the patient therefore avoiding the need for immunosuppression [218,219]. Furthermore, there has been a strong push in Japan and the USA to create a global library of iPSC lines from donor somatic cells that are homozygous at several gene loci to match a patient’s individual HLA type, thus making grafts without immunosuppression possible [218,219]. While iPSCs introduce exciting possibilities and advancements in regenerative medicine, their use still presents potential difficulties. When considering using autografts in clinical cases of SCI, the ideal time for transplantation is within two to four weeks post injury yet the generation and induction of iPSCs towards a neural lineage takes at least four to six months, with another year necessary for quality control [219]. The method of reprogramming can also be of concern due to the use of various viruses, possibility for mutagenesis and transgene reactivation [218,219]. There is also an argument that iPSCs never truly become “blank” stem cells and instead retain an epigenetic “memory” of their tissue source, which can then possibly influence the nuances of differentiation and final phenotype [220,221]. Finally, while exciting, iPSCs are a technology in its infancy that does not have decades of research supporting it as with MSCs or ESCs; many questions remain to be answered.

### 4.2. Differentiation of Induced Pluripotent Stem Cells (iPSCs)

A chief advantage of iPSCs technology is that iPSCs can be directed into the three main neural types *in vitro*. A major effort has been put forth to develop reliable iPSCs differentiation protocols that consistently generate functional neurons and glial cells so as to study the differences in neurons and neuronal networks in both healthy and impaired states [222,223,224,225,226,227,228,229,230,231].

In one protocol, human iPSCs were driven towards a neuroepithelial lineage using exogenous patterning molecules. The presence of mitogens allowed for the generation of astroglial progenitors, which could then be differentiated into functional astrocytes via ciliary neurotrophic factor [232]. In another protocol, mouse and rat fibroblasts were directly reprogrammed into oligodendrocyte precursor cells via forced expression of Sox10, Olig2, and Zfb536. These precursors exhibited expected morphologies and gene expression and gave rise to mature oligodendrocytes that could ensheath dorsal root ganglion cells *in vitro* and form myelin *in vivo* [233]. In another study reporting on the creation of neurons from iPSCs, the overexpression of Neurogenin-2 quickly and efficiently transformed iPSCs into neurons that formed spontaneous excitatory synaptic networks, which exhibited plasticity and synaptically integrated once transplanted into the mouse brain [234]. Another report showed that adult human fibroblasts could directly be reprogrammed into functional neurons that formed synapses via a cocktail of miR-124, BRN2, and MYT1L [235]. In addition to the aforementioned cell types, other groups have shown that iPSCs can be successfully driven towards glutamatergic, GABAergic, motor, and retinal neuron phenotypes, amongst others [236,237,238,239,240,241,242,243,244,245,246,247,248]. While not specific to SCI, these results demonstrate that developing differentiation protocols that generate specific neural subtypes can open up new avenues in understanding and creating therapies for neuropathologies.

Currently, preclinical studies have used iPSC technology and differentiation strategies for disease modeling *in vitro* and *in vivo*. Despite being a young technology, some of these have already eventuated into clinical trials or are in progress to evaluate the safety and efficacy of iPSCs. Various studies are exploring a variety of degenerative states such as macular degeneration, recessive dystrophic epidermolysis bullosa, Parkinson’s disease, thrombocytopenia, Multiple Sclerosis, spinal cord injury, corneal endothelial dysfunction, Stevens-Johnson syndrome, heart failure, retinitis pigmentosa, and refractory thrombocytopenia ([219] and ref therein). Not surprisingly, considering the substantial lack of preclinical and clinical stem cell transplantation studies following cervical SCI, there are no clinical studies that examine iPSC transplantation in treating cervical SCI.

### 4.3. Treating Cervical SCI with iPSC Technologies

The cervical spinal cord contains the long tracts connecting the rostral and caudal portions of the CNS, as well as the sensory and motor neurons for upper limb function and diaphragm-mediated respiration [249]. Despite the fact that SCI in the cervical region accounts for more than half of all presented cases, there is a tremendous lack of research to date exploring potential targeted therapies for this region. To date, there are currently four preclinical studies looking at iPSCs transplantation within the cervical cord.

One study using iPSCs in a cervical SCI model examined transplantation within what could be described as an “early chronic” window. The majority of transplantation studies utilize an acute injury model, transplanting by two weeks post SCI as this has been empirically determined to be the ideal clinically relevant time point [219]. However, acute injury models do not assist patients living with chronic SCI. Nutt and colleagues (2013) developed an early chronic injury model that mimics the deficits seen in humans using rats [250]. Four weeks following a cervical contusion injury at C4, rats received intraspinal transplantations of iPSC-derived neural progenitor cells and fibroblast rostral and caudal to the lesion site. By eight weeks post-transplant, NeuN/FOX-3 labeling showed a portion of transplanted cells differentiated into mature neurons. Despite intermingling between transplanted cells and host neurons, transplanted cells did not express glutamate receptors. Additionally, transplanted cells did not express serotonin but were positive for GABA and were shown to localize with host positive choline acetyltransferase. Behaviorally, grasping and weight bearing were only slightly improved by the transplants.

Another study by Li and colleagues (2015) evaluated respiratory function following iPSC-derived astrocyte transplants (following a standard differentiation protocol and also engineered to overexpress GLT1 [251]. In this work, both rats and mice underwent a C4 contusion injury resulting in chronic diaphragm dysfunction and phrenic motor neuron deterioration. Immediately post injury, they received an intraspinal transplant comprised of two separate injections rostral and caudal to the lesion and within the ventral horn. At two day, two week, and four week post injury/transplant time points, grafts survived and differentiated into astrocytes (GFAP positive), did not display any tumorigenicity, and had less than 10% proliferation (Ki67 staining). In addition, following transplant of GLT1-overexpressing astrocytes, lesion area and total lesion volume were reduced within one millimeter rostral and caudal to the lesion epicenter and preserved innervation of the diaphragm neuromuscular junction. By analyzing spontaneous EMG activity, it was also demonstrated that GLT1-overexpressing astrocyte transplants significantly increased EMG amplitude in the dorsal region of the hemi diaphragm, thus indicating preservation of diaphragmatic respiratory function.

Lu and colleagues (2014) examined the effect of sub-acute human iPSC-derived neural stem cells (NSCs) transplantation from an 86-year-old male in a C5 lateral hemisection in a rat model [252]. While not extensively characterized, the NSCs *in vitro* displayed a dramatic reduction in Tra1-81 and SSEA4 (pluripotency markers), and maintained expression of Nestin and Sox2 (NSC-associated markers). These NSCs were embedded in a fibrin matrix alongside a cocktail of growth factors and administered via intraspinal transplantation two weeks post injury. By three months post transplantation, grafts had survived and distributed throughout the lesion. The majority of grafted cells expressed NeuN (neuronal specific nuclear marker), but rarely doublecortin (NSC marker). Mature neuronal markers MAP2 and Tuj1 were expressed along with mature astrocytic marker GFAP, suggesting favored differentiation into neuronal and astrocytic lineages. There was also evidence of a small percentage of grafted cells expressing ChAT (characteristic of spinal motor neurons) and expressing Ki67, which is indicative of proliferation within the graft. No grafted cells expressed the serotonergic marker 5-HT. Most notably in this study was the robust axonal outgrowth of grafted cells throughout the entirety of the host CNS (from the lesion site rostral to the olfactory bulbs and caudally to lumbar spine sections). In addition, there was evidence of integration of host axons into the grafted lesion site. Despite these interesting results, no behavioral recovery was observed.

Kobayashi and colleagues (2012) chose to examine the safety and efficacy of sub-acute transplantation of iPSC-derived neural stem cell transplants following cervical SCI in a non-human primate model [253]. Human iPSCs were cultured and neuronally induced to form neurospheres. Primary neurospheres were passed into secondary and tertiary neurospheres prior to transplantation. Adult female marmosets were given a moderate contusion at the C5 level and 9 days later received an intraspinal injection of cultured iPSC-neurospheres in the lesion epicenter. By 12 weeks post-transplant, hematoxylin-eosin staining revealed that the grafted cells survived and differentiated into all 3 neural subtypes (NeuN, GFAP, Olig1); the transplanted animals displayed a significant difference in cystic cavity size and no evidence of tumorigenicity was found in any of the animals. Undifferentiated iPSCs stained positive for Oct4, while cells that were positive for HNu did not. While severe demyelination was evident surrounding the lesion site in both transplanted and control groups, quantification revealed significantly higher degrees of demyelination in the control group at 12 weeks post-transplant. In accordance with these findings, conventional MRI and Myelin-mapping revealed more myelin sparing in the transplanted group and an intramedullary high-signal intensity area in the lesion site of the control group. Grafts had a higher number of neurofilaments and descending motor axons at the lesion center than the control group. Furthermore, this was coupled with evidence of increased angiogenesis, as demonstrated by staining of platelet endothelial cell adhesion molecule-1. Calcitonin generated peptide fibers, which are involved in spinal pain mechanisms, did not differ between transplanted and control groups. Behaviorally, contusion at the C5 level led to tetraplegia in the marmosets with a gradual improvement in motor function, as was expected in a severe central cord injury model. By eight weeks following SCI, significant differences in the open field test were found between transplanted and control groups, which stayed consistent throughout the study. Additionally, the bar grip strength and cage climbing tests were also found to be statistically significant between the groups by eight weeks post-transplant.

## 5. Conclusions and Future Considerations

Cervical-level SCIs account for the majority of presented cases and place a formidable lifetime financial burden on the nation. Despite the increase in patient survival rates due to improvements in modern medicine, there has been little advancement in ameliorating subsequent deficits in respiratory and motor dysfunction and quality of life still remains poor. Therapies that aim to restore function, even partially, are urgently needed and would make a substantial impact in helping patients regain independence. Cell transplantation therapies in SCI seek to accomplish this by increasing the innate host regenerative capacities through donor-secreted trophic and immunomodulatory factors, providing scaffolding to bridge the lesion site to promote regeneration of native axons and restoration of rostral and caudal circuitry, and by replacing SCI-lost neurons and glia.

Stem cell transplantations are a logical step in achieving this as they have the ability to replace specific phenotypes within the lesion and can evoke a strong immunological, regenerative, and healing response from the host. MSC and ESC-derived grafts for treatment of cervical SCI have been found to positively influence the inhibitory environment of the lesion and have occasionally assisted in behavioral recovery. However, these cell types raise concerns that make their use in commercial and clinical applications difficult. MSCs are limited by their potency, transient presence following transplantation, and functional outcomes. ESCs are subject to ethical constraints, carry karyotypic abnormality and tumorigenicity risks, and require immunosuppression. The use of iPSCs circumvents some of the pitfalls of MSCs and ESCs, namely potency in conjunction with source. The use of reprogrammed cells is a developing technology and not without its own concerns though; reprogramming methods can be inefficient and difficult to safely clinically translate, iPSCs are subject to karyotypic instability and may retain an epigenetic memory, and there is simply not an abundance of preclinical and clinical data.

To date, there is only a limited pool of research examining iPSC-derived transplants in SCI—even fewer of those research studies being specific to cervical injury. Current research predominantly uses rat animal modeling, and in one case, non-human primates. In these studies there was evidence of transplanted cell survivability, differentiation into mature neurons (conflicting evidence exists regarding synaptic activity of these mature neurons), intermingling between transplanted cells and host neurons, reduced lesion area and volume, increased angiogenesis, no tumorigenicity, and amelioration of motor deficits.

These results are certainly promising, especially when considered in conjunction with the positive findings associated with alternative stem cell-derived transplants. However, there still exists a significant research gap in this area, and underlies a major need to identify, examine and develop clinically relevant cervical SCI therapeutics. With so few studies existing using iPSC technology at the cervical level, it is difficult to speculate as to what factors would improve the efficacy of transplantation. At this current time, it may be of value to consider the experimental design of studies in this niche to have better clinical translation. The use of appropriate animal models, inherent capabilities of the transplanted cell type, timing of transplantation, and the chosen behavioral assessments to assess functional recovery have importance.

Experimentally, it is important to choose an anatomically appropriate model that mimics human cervical SCI pathology and motor deficits. The authors recognize that no individual animal model will completely embody every human clinical manifestation of cervical SCI, however, it is worthwhile to be able to recognize the strengths and weaknesses of each type within the context of achieved results. In rodent species, rats are superior to mice in that a contusion injury will form cystic cavitation that resembles what is seen in humans while mice develop matrices of connective tissue instead [254,255,256]. Furthermore, rats are able to perform detailed tasks predominantly using the biceps and triceps brachii and extensor and flexor carpi radialis muscles of the forelimbs and digits—an ability that is eviscerated by damage to the descending tracts following cervical injury, much as in humans. The usage of non-optimal behavioral tasks and assessments (or relying on a singular task) may confound what is defined as “functional recovery”. Rats are cost-effective, easily trainable, and mimic some of the anatomical and motor deficits seen in clinical presentations of cervical SCI, however, the corticospinal tract projection is predominantly located in the dorsal column and decussates at the brainstem meaning that axons will have derived from the contralateral motor cortex. In contrast, the location of the primate’s corticospinal tracts are predominantly in the dorsolateral column and experience many decussations over the spinal midline indicating that axons will have derived from both the left and right motor cortex [72]. This is significant because it means that following cervical SCI, primates may be more adept at circuitry rewiring and restoration than rats. Therapies that achieve limited or no functional recovery in rodents may have more potent effects on primates.

Additionally, the predominant derivation of transplanted iPSCs populations has been neural progenitors (NPCs). NPCs certainly show survivability, integration and colocalization with host cells, evidence of differentiation via the presence of mature markers, and are associated with improved functional outcomes. However, they are often transplanted as uncharacterized or mixed populations, which limit experimental control and clinical translation. Once transplanted, differentiation and phenotype are beyond the experimenter’s control. Restoring circuitry within the injured cord requires that host axons are able to overcome the inhibitory environment of the lesion and reconnect or that transplanted cells within the lesion can form a relay system to connect the rostral and caudal CNS. It may be relevant to transplant cells that are further matured towards a neuronal lineage to accomplish this *versus* NPCs that will predominantly yield supporting glial phenotypes.

The use of iPSCs represents an exciting interface between disease modeling, developmental biology, and regenerative medicine. Treating cervical SCI with iPSCs is currently limited by a significant lack of published studies despite its overwhelming clinical relevance. Furthermore, discrepancies within experimental design may contribute to confound results. As iPSC technology increases and knowledge of differentiation protocols progress, it is our belief that iPSC-derived transplants (possibly in conjunction with combinatorial treatments) will provide an encouraging avenue to positively address the unique and multifaceted requirements triggered by cervical SCI.
